# A mobile phone based tool to identify symptoms of common childhood diseases in Ghana: development and evaluation of the integrated clinical algorithm in a cross-sectional study

**DOI:** 10.1186/s12911-018-0600-3

**Published:** 2018-03-27

**Authors:** Konstantin H. Franke, Ralf Krumkamp, Aliyu Mohammed, Nimako Sarpong, Ellis Owusu-Dabo, Johanna Brinkel, Julius N. Fobil, Axel Bonacic Marinovic, Philip Asihene, Mark Boots, Jürgen May, Benno Kreuels

**Affiliations:** 1 Division of Tropical Medicine, First Department of Medicine, University Medical Center Hamburg-Eppendorf (UKE), Hamburg, Germany; 2 Infectious Disease Epidemiology, Bernhard Nocht Institute for Tropical Medicine (BNITM), Hamburg, Germany; 3 Kumasi Center for Collaborative Research in Tropical Medicine (KCCR), College of Health Sciences, Kwame Nkrumah University of Science and Technology (KNUST), Kumasi, Ghana; 4 Department of Public Health Medicine, School of Public Health, University of Bielefeld, Bielefeld, Germany; 5 School of Public Health, University of Ghana, Accra, Ghana; 6 National Institute for Public Health and the Environment (RIVM), Bilthoven, The Netherlands; 7 Viamo, Accra, Ghana

**Keywords:** mHealth, Algorithm, Symptom assessment, Decision making, computer assisted, Interactive voice response, Africa, Children

## Abstract

**Background:**

The aim of this study was the development and evaluation of an algorithm-based diagnosis-tool, applicable on mobile phones, to support guardians in providing appropriate care to sick children.

**Methods:**

The algorithm was developed on the basis of the Integrated Management of Childhood Illness (IMCI) guidelines and evaluated at a hospital in Ghana. Two hundred and thirty-seven guardians applied the tool to assess their child’s symptoms. Data recorded by the tool and health records completed by a physician were compared in terms of symptom detection, disease assessment and treatment recommendation. To compare both assessments, Kappa statistics and predictive values were calculated.

**Results:**

The tool detected the symptoms of cough, fever, diarrhoea and vomiting with good agreement to the physicians’ findings (kappa = 0.64; 0.59; 0.57 and 0.42 respectively). The disease assessment barely coincided with the physicians’ findings. The tool’s treatment recommendation correlated with the physicians’ assessments in 93 out of 237 cases (39.2% agreement, kappa = 0.11), but underestimated a child’s condition in only seven cases (3.0%).

**Conclusions:**

The algorithm-based tool achieved reliable symptom detection and treatment recommendations were administered conformably to the physicians’ assessment. Testing in domestic environment is envisaged.

**Electronic supplementary material:**

The online version of this article (10.1186/s12911-018-0600-3) contains supplementary material, which is available to authorized users.

## Background

Despite a reduction of childhood mortality by 53% since 1990, almost six million children under 5-years died in 2015 [1]. High child mortality rates are observed especially in sub-Saharan Africa, where 83 out of 1000 live births die, mostly due to acute respiratory infections, diarrhoeal disease and malaria [2]. Timely diagnosis and treatment of diseases are life-saving, yet provision and access to health-care services are still limited in many areas of sub-Saharan Africa [3]. Long distances to health care facilities have a strong influence on health-seeking behaviour and many deaths could be prevented if adequate treatment was initiated earlier [4, 5].

To improve diagnosis and treatment for children living in rural and remote areas, innovative strategies are needed. mHealth (mobile Health), the use of mobile devices to support public health measures, offers great potential to enhance communication between patients and the professional health care system [6, 7]. Mobile phone targeted health interventions have increasing potential in many developing countries, since the number of mobile phone subscriptions is constantly growing and the mobile phone network is also expanding to rural areas [8].

The idea of this study was the development of an mHealth tool to support guardians of children living in areas with limited access to health care. To achieve this goal, the Integrated Management of Childhood Illnesses (IMCI) guidelines [9] were used to develop a clinical algorithm to assess disease symptoms in sick children that could be implemented into an interactive voice response (IVR)-system applicable in automated health hotlines. The transfer of information would rely on audio files - rather than on text messages - which could be useful for the multitude of illiterates in sub-Saharan Africa [10]. At the beginning of the development and repeatedly every 6 months during the consecutive process of testing, data analysis and writing of the manuscript (last search on 13.09.2017) we conducted a systematic literature search on PubMed to identify studies that had evaluated mHealth tools in African settings.

So far, the systematic literature research has not revealed any studies using algorithm based IVR-systems for disease detection in children [7, 11, 12]. Some studies have been conducted in which the IMCI guidelines were translated into mHealth interventions to assist health care workers. In one study by Ginsburg et al. a mHealth application, integrating a digital version of the IMCI guidelines with a breath counter and a pulse oximeter, was developed to support health care workers in the diagnosis of pneumonia in children in Ghana [13]. The authors concluded that their tool had “the potential to facilitate prompt diagnosis and assessment” [14]. In another study by Rambaud-Althaus et al. the authors developed a new algorithm for the management of children under 5 years of age living in resource poor settings. It included point-of-care tests and clinical predictors for acute illnesses to improve the rational use of antimicrobials [15]. In a subsequent controlled non-inferiority study in Tanzania they concluded that the use of their algorithm “improved clinical outcome and reduced antibiotic prescription” [16]. However, these studies aimed at health care workers, whereas our study directly targets guardians of sick children.

This study was part of the electronic Health Information and Surveillance System project (eHISS) that aimed at conceptualizing and piloting a mobile phone based tool to collect individual disease information and to provide corresponding treatment recommendations. Health information from participating populations and the simultaneous collection of spatio-temporal data on the incidence of fever, diarrhoea and respiratory distress were envisaged for monitoring potential disease outbreaks. This manuscript presents the development and evaluation of an algorithm based tool to identify symptoms of common childhood diseases and to provide basic treatment recommendations, focussing on testing its medical correctness. User experiences with the finalized tool were evaluated in another study [17].

## Methods

### Decision making process

An expert panel consisting of clinicians, epidemiologists/biostatisticians, public health experts and communication researchers was involved in the development of the clinical algorithm and its translation into an IVR-system. The panel met three times over a period of 2 years to discuss the draft versions of the system and to decide on necessary adjustments using a Delphi method-like approach. A detailed description of the panel’s composition and the methods how the clinical algorithm and IVR-system were developed, amended and evaluated (further evaluation results are also published elsewhere [18]) is given in the supplementary files (see Additional file 1).

### Development of the algorithm

The IMCI guidelines served as a template representing the standard of care for diagnosis and management of childhood illnesses in primary health care in resource poor settings. Furthermore, they give concise instructions on how to manage the most common childhood diseases. After assessing a child’s condition (“ask, look, feel”), the IMCI’s flowchart will guide the user to a disease, assign it to a respective triage level (immediate referral, management in the outpatient facility or home management) and give instructions for treatment [9]. To apply the IMCI guidelines to the target population (lay people vs. medical personnel) and the field of application (home use in IVR-system vs. health care setting), it was necessary to make some changes in the IMCI flowchart. As the IMCI guidelines were developed for medical personnel, they needed to be amended to ensure comprehension by lay people while gaining the maximum possible amount of information about a child’s health status. Therefore, we used only questions that could be answered without pre-existing medical knowledge and did not require a clinical inspection and examination. The panel excluded questions that included physical examination (e.g. counting the breaths or identifying oedema), since we could not expect to get reliable results from untrained people. It allowed asking for symptoms (i.e. fever, cough, diarrhoea, vomiting), the existence of dangerous symptoms (“danger signs”) and further questions to assess the level of respiratory infection and dehydration. Diseases and their respective symptoms were included and excluded based on the prevalence of symptoms as determined by a survey of the hospital’s record-book that was analysed for a year preceding the study and with data on diagnoses from ongoing studies on disease aetiology. The main diagnoses made in the study hospital (multiple diagnoses possible) are *Plasmodium falciparum* malaria (59%), pneumonia and other respiratory tract infections (33%), gastrointestinal infection (17%), and bloodstream infections (5%) (unpublished data). The IMCI’s three triage levels for respective diseases remained the same, only the advice was refocused for home use (e.g. presentation to a hospital immediately/in 24 h (algorithm) vs. referral or management in the outpatient facility (IMCI)). After this modification process the panel determined the order of questions. The IMCI guidelines’ hierarchic structure was kept by assessing the severity of a confirmed symptom with additional questions.

The algorithm’s tasks were to identify symptoms of common childhood illnesses (symptom detection), to detect an illness (disease assessment) as well as to estimate the severity of the symptoms and give suitable advice (treatment recommendation). These three tasks were to be compared to the physicians’ findings and recommendations.

**Fig. 1:**
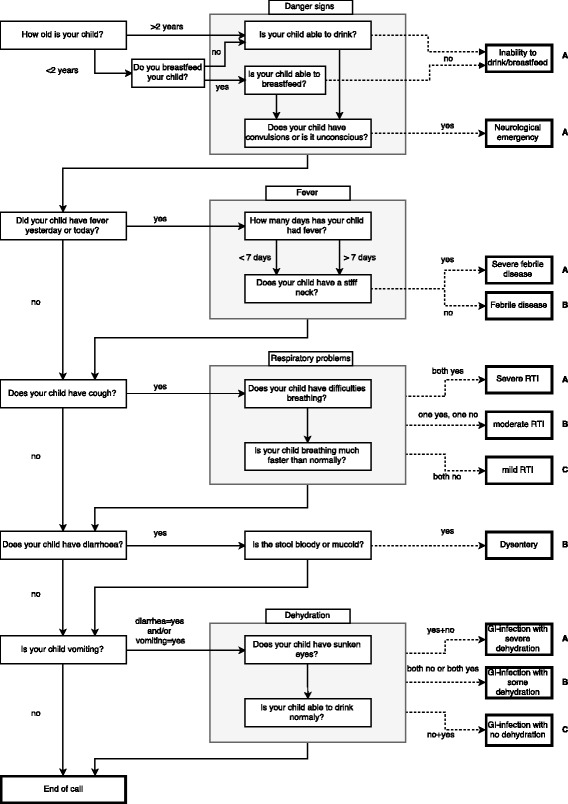
Flowchart of the clinical algorithm. Solid arrows show the flow from question to question. Unless otherwise indicated (e.g. arrow marked with “yes”), the next question was asked irrespective of the answer to the previous question. Dashed arrows indicate the disease assessments (shown in double framed boxes) resulting from answers to specific questions. Each disease assessment is associated with a treatment recommendation, indicated as either A (requiring emergency treatment), B (requiring causal treatment), or C (requiring home care). Grey boxes indicate contextually related questions (e.g. all questions relating to respiratory problems)

The finalized clinical algorithm was based on dichotomous questions asked successively as shown in Fig. 1. The first questions referred to the child’s age and the existence of “danger signs” such as inability to drink or breastfeed and neurological emergencies (e.g., convulsions and unconsciousness). The following questions aimed at identifying the symptoms fever, cough, diarrhoea and vomiting. Whenever the guardians responded that their child suffered from one of these symptoms, more specific questions (highlighted with grey background) were asked to assess symptom severity. Based on the symptom severity, calls were assigned to a treatment recommendation: A = requiring emergency treatment, B = requiring causal treatment or C = requiring home care. Additional advice was given on how to manage the child on the way to the hospital (e.g., “offer enough to drink”) or on how to treat the child at home (e.g., advise on the use of oral rehydration salts, if applicable). The algorithm was designed to register information on multiple symptoms for one participant but assigned calls to only one treatment recommendation according to the most life-threatening condition.

The following disease assessments with different severity levels were included in the algorithm: *inability to drink or breastfeed*; *neurological emergency*; *severe febrile disease*; *febrile disease*; *respiratory tract infection* (RTI) categorized as *severe*, *moderate* or *mild*; *gastrointestinal infection* (GI-infection) with *severe*, *moderate* or *no dehydration*; and *dysentery.*

### Development of the IVR-system

The users’ requirements concerning the application of a mobile phone based IVR-system for seeking health care were investigated by ascertaining the caregivers’ attitudes, their motivations for adoption and the barriers to its implementation in focus group discussions [18]. Data from this analysis was used to implement the clinical algorithm in an IVR-system (the IVR-system incorporating the clinical algorithm will be referred to as the “tool”). Based on their experience in working with mothers in the area, clinicians translated the questions and recommendations into the commonly spoken language Twi in the manner they would ask these questions during medical consultations and recorded them as audio files. VOTO Mobile (now called Viamo [19]), a Ghana-based company specializing in interactive SMS (short message service) or voice calls [19], developed and operated the IVR-system. Users started consulting the tool by calling a hotline. After hearing the audio files, users answered the questions by pressing number codes and were thereby (navigated) guided through the algorithm’s questions. Subsequently, the tool was tested by participants and challenges in the usage of the tool were assessed in further focus group discussions. The usability (System Usability Scare median: 79.3; range: 65.0–97.5) was rated acceptable [17]. Additionally, the tool was piloted with 50 target users to make sure that the audio files and the respective questions are really understood. All participants could understand the questions and were able to use the tool via mobile phone.

#### Study area and participants

This cross-sectional study was conducted at the Agogo Presbyterian Hospital (APH) in Ghana between October 2014 and January 2015. APH is a district hospital and provides beds for about 250 patients. The study was conducted in the children’s outpatient department that is frequently visited by families for both emergency and follow-up treatment.

Guardians were recruited to the study if they were ≥18 years old, accompanying a sick child (≥1 month and < 15 years), fluent in the local language Twi and if they had not come for referral treatment or review. Ethical approval for this study was obtained from the Kwame Nkrumah University of Science and Technology (KNUST) in Kumasi, Ghana (Reference number: CHRPE/AP/278/14).

#### Study procedures

Guardians were recruited in the OPD before seeing the attending physician. Study procedures were explained and written informed consent was obtained from the guardians. Study personnel instructed the participant on how to use the tool before the participant conducted the phone call and completed the tool’s questions. After finishing the phone call, the participant consulted the physician, who wrote a report into the patient file. The patient file was reviewed and a member of the study team, who was blinded to the results from the tool assessment, transcribed symptoms, diagnoses, treatment procedures and possible reassessment recommendations on a questionnaire.

#### Data analysis

The tool’s performance against the medical records was evaluated by measuring the inter-rater agreement using Cohen’s Kappa and corresponding *p*-value. The Kappa values were interpreted as suggested by Landis and Koch [20]. Other calculated values were sensitivity, specificity, positive predictive value (PPV) and negative predictive value (NPV). As this was an explorative study, sample size was not based on a formal calculation, but determined by the number of participants that could be recruited within the planned recruitment period of 3 months. To achieve reliable results it was intended to recruit at least 200 participants.

The tool’s outcome was compared to the physicians’ findings (used as gold standard) in three categories: symptom detection, disease assessment and treatment recommendation. While the detected symptoms could be compared directly to the respective physicians’ notes, variables had to be created to evaluate disease assessment and treatment recommendation. Concerning disease assessment, the tool’s findings (e.g., *severe RTI*) were compared to variables composed of physicians’ assessment (e.g., tachypnoea) or diagnoses (e.g., pneumonia). Concerning treatment recommendations, three variables were defined, composed of the physicians’ assessment, treatment or advice for re-evaluation. These three variables allowed comparison to the tool’s treatment recommendations (A1–3), determining the need for emergency, causal or home treatment, as shown in Table 1. Data were analysed using Stata 14 (College Station, TX: StataCorp LP).

**Table 1:** Comparing the tool’s treatment recommendation to the physicians’ assessment

Triaged as	Tool	Physicians’ notes
A	Requiring emergency treatment: “Take your child to the nearest hospital immediately!”	Emergency treatment (e.g. i.v. fluids, i.v. antibiotics, i.m./i.v. antimalarial drugs) or hospitalization for further investigation
B	Requiring etiologic treatment: “Take your child to the nearest hospital within 24 h!”	Etiologic treatment (e.g. antibiotics, antimalarial drugs in oral form etc.) or follow-up
C	Requiring home care: “Treat your child at home and assess disease progression carefully!”	Symptomatic treatment (e.g. nasal drops or paracetamol) or follow-up was not expected to be needed

## Results

### Study participants

A total of 294 participants were recruited for this study. Of these, 57 (19.4%) were excluded from the analysis as they did not complete the algorithm (*n* = 27, 9.2%), contained missing data in their hospital files (*n* = 20, 6.8%) or did not show any symptoms considered by the tool (*n* = 10, 3.4%), namely conjunctivitis, constipation, polydactyly, seborrheic dermatitis, pruritus and vaginal bleeding. Finally, data from 237 participants were included into the study.

The median age of the guardians was 31 years (range: 18–71, interquartile range: 24–36) and the vast majority was female (*n* = 227, 95.8%). The median age of the children was 3 years (range: 0–14, interquartile range: 1–4). Most children (*n* = 213, 89.9%) were accompanied by their biological parents. In total, 192 (81.0%) participants reported to use a mobile phone regularly.

### Evaluation of the tool

#### Symptom detection

The highest agreement between the findings of the tool and the physician was observed for cough (percentage agreement 82.3%, kappa = 0.64, *p* < 0.01, sensitivity = 91.5%, NPV = 87.4%). Good agreement was also seen for fever (83.5%, kappa = 0.59, p < 0.01, sensitivity = 90.4%, NPV = 74.6%) and diarrhoea (84.4%, kappa = 0.57, *p* < 0.01, sensitivity = 86.1%, NPV = 96.5%), while vomiting showed reasonable agreement (76.4%, kappa = 0.42, *p* < 0.01, sensitivity = 67.2%, NPV = 88.2%, Table 2).

**Table 2:** Comparison of the tool and the physicians in terms of symptom detection

Symptom	Agreement (%)	Cohen’s kappa	*p*-value ^a^	Sensitivity (%)	Specificity (%)	PPV ^b^	NPV ^c^	Prevalence (%)
Fever	83.5	0.59	< 0.01	90.4	67.1	86.8	74.6	70.5
Cough	82.3	0.64	< 0.01	91.5	71.0	79.3	87.4	54.9
Diarrhoea	84.4	0.57	< 0.01	86.1	84.0	54.4	96.5	18.1
Vomiting	76.4	0.42	< 0.01	67.2	79.3	51.3	88.2	24.5

^a^*p*-value for Cohen’s Kappa

^b^PPV = positive predictive value

^c^NPV = negative predictive value

#### Disease assessment

As shown above, the algorithm derived 11 disease assessments for the most common childhood diseases. The physicians neither diagnosed inability to drink/breastfeed and stiff neck, nor symptoms determining severe febrile disease in any of the patients. Thus, nine out of 11 disease assessments were available for data analysis. The results are displayed in Table 3.

**Table 3:** Accordance of the tool’s disease assessments and the physicians’ findings ordered to the respective treatment recommendation

	Agreement (%)	Cohen’s kappa	*p*-value^a^	Sensitivity (%)	Specificity (%)	PPV^b^	NPV^c^	Prevalence^d^ (%)
A								
Neurological emergency	77.2	− 0.01	0.71	0	77.5	0	99.5	0.4
Severe RTI^e^	84.0	0.3	< 0.01	54.6	87.0	30.0	94.9	9.3
GI-infection^f^ with severe dehydration	93.3	0.08	0.09	16.7	95.2	8.3	97.8	2.5
Inability to drink/breastfeed	n/a	n/a	n/a	n/a	n/a	n/a	n/a	n/a
Severe febrile disease	n/a	n/a	n/a	n/a	n/a	n/a	n/a	n/a
B								
Febrile disease	78.9	0.51	< 0.01	83.2	68.6	86.3	63.2	70.5
Moderate RTI^e^	63.7	0.05	0.21	27.3	77.8	32.1	73.5	27.9
GI-infection^f^ with some dehydration	81.9	0.25	< 0.01	66.7	82.9	20.8	97.4	6.3
Dysentery	84.0	0.17	< 0.01	71.4	84.3	12.2	99.0	3.0
C								
Mild RTI^e^	62.5	−0.07	0.87	17.0	75.5	16.7	76.0	22.4
GI-infection^f^ with no dehydration	73.4	0.2	< 0.01	27.4	89.7	48.6	77.7	26.2

A = requiring emergency treatment

B = requiring causal treatment

C = requiring home care

^a^*p*-value for Cohen’s Kappa

^b^PPV = positive predictive value

^c^NPV = negative predictive value

^d^Prevalence of the disease assessments made by the physicians

^e^RTI = respiratory tract infection

^f^GI-infection = gastrointestinal infection

The five most common physicians’ diagnoses were *febrile disease* (*n* = 167, 70.5%), *moderate RTI* (*n* = 66, 27.9%), *GI-infection with no dehydration* (*n* = 62, 26.2%), *mild RTI* (*n* = 53, 22.4%) and *severe RTI* (*n* = 22, 9.3%). *GI-infection with some dehydration* (*n* = 15, 6.3%), *dysentery* (*n* = 7, 3.0%), *GI-infection with severe dehydration* (n = 6, 2.5%) and *neurological emergency* (n = 1, 0.4%) were less often diagnosed.

Good agreement was observed *for febrile disease* (78.9%, kappa = 0.51, *p* < 0.01, sensitivity = 83.2%, NPV = 63.2%), while fair agreement was seen for *severe RTI* (84.0%, kappa = 0.3, p < 0.01, sensitivity = 54.6%, NPV = 94.9%) and *GI-infection with some dehydration* (81.9%, kappa = 0.25, p < 0.01, sensitivity = 66.7%, NPV = 97.4%). *GI-infection with no dehydration* (73.4%, kappa = 0.2, *p* < 0.01, sensitivity = 27.4%, NPV = 77.7%) showed slight agreement. The highest percentage-agreement was noted for *GI-infection with severe dehydration,* even though with a low kappa, as this condition was diagnosed by the physicians very rarely (93.3%, kappa = 0.08, *p* = 0.1, sensitivity = 16.7%, NPV = 97.8%). The algorithm detected five out of seven cases of *dysentery* entailing fair agreement (84.0%, kappa = 0.17, p < 0.01, sensitivity = 71.4%, NPV = 99.0%).

The physicians noted one case of convulsions, which was not detected by the tool. Nevertheless, 53 participants misdiagnosed their children with convulsions or unconsciousness resulting in a poor agreement for *neurological emergency (77*.2%, kappa = − 0.01, *p* = 0.71, sensitivity = 0%, NPV = 99.5%).

#### Treatment recommendation

As shown in Table 4, the physicians ranked most of the children as B (requiring causal treatment [*n* = 175, 73.8%]), followed by A (requiring emergency treatment [*n* = 48, 20.3%]) and C (requiring home care [*n* = 14, 5.9%]), while the tool assessed the majority of patients as A (*n* = 171, 72.2%), followed by B (*n* = 55, 23.2%) and C (*n* = 11, 4.6%). The tool detected 42 out of 48 cases that were assessed as A by the physician (87.5%) and ranked the six (12.5%) remaining cases as B. These six cases were severe forms of malaria and sepsis requiring hospitalization. The tool ranked them as B due to the disease assessment *febrile disease*.

**Table 4:** Accordance of the tool and the physicians in terms of treatment recommendation

	Physicians’ assessment			Total
Tool’s treatment recommendation	A	B	C	
A	42	121	8	171
B	6	47	2	55
C	0	7	4	11
Total	48	175	14	237

A = requiring emergency treatment

B = requiring causal treatment

C = requiring home care

Out of the 175 physician-ranked B-cases, 47 cases (26.9%) were correctly identified as B by the tool, 121 patients (69.1%) were ranked as A and 7 (4.0%) as C. The tool underestimated the latter cases as *mild RTI* or *GI-infection with no dehydration*, but these patients received causal treatment (e.g., with antibiotics) by the physicians or were asked to present for reassessment on the following days. Among the 14 physician-ranked C-cases, four were correctly assessed, (28.6%) correctly, however two (14.3%) were declared as B and eight (57.1%) as A.

Overall, data analysis indicated a weak agreement (39.2%, kappa = 0.11, *p* < 0.01) between the tool’s treatment recommendation and the physicians’ assessment. Nevertheless, as described above, underestimation of disease severity was low. Only seven out of 237 cases (3.0%) would have been ranked as C although they needed to be examined by a physician (either A or B by the physician). Furthermore, the accordance of tool and physicians’ assessment, whether a child should be presented at a hospital (A or B) or not (C), showed strong agreement (92.8%) with fair kappa values (0.28).

## Discussion

This study describes the development and evaluation of an algorithm-based IVR-tool for guardians of sick children in sub-Saharan Africa. It was shown that, compared to the subsequent examination by attending physicians, the tool performed well in identifying the three leading disease symptoms fever (agreement = 83.5%, kappa = 0.59), cough (agreement = 82.3%, kappa = 0.64), and diarrhoea (agreement = 84.4%, kappa = 0.57). The detection of vomiting was moderate (agreement = 76.4%, kappa = 0.42). Additionally, the tool was able to give out appropriate treatment recommendations. Merely seven out of 237 patients (3.0%) were not sent to the hospital by the tool, although physicians applied causal treatment for specific illnesses (e.g. prescribed antibiotics). These results indicate that the tool can provide basic treatment recommendations without a medical health care professional nearby, although disease assessment was not achieved in a reliable way. The discrepancy between adequate treatment recommendations and inadequate disease assessment may be explained by the registration of multiple disease assessments but only one treatment recommendation. Guardians were not able to answer the more specific questions correctly after confirming a symptom, but they seemed to be able to estimate the need for treatment. This was shown by the tool’s good performance in deciding whether or not a child has to be presented at a hospital (agreement = 92.8%, kappa = 0.28). Thus, this tool could contribute to timely diagnosis and treatment and may be effective in lowering childhood mortality in areas with limited access to health care services.

Success and failure of this tool are strongly dependent on the guardians’ ability to correctly assess their children’s health status. A systematic review by Geldsetzer et al. evaluated the guardians’ recognition of childhood illnesses in developing countries. Due to low values of sensitivity for the recognition of fever, diarrhoea and pneumonia, the authors concluded that “survey data based on these reports should not be used for disease burden estimations” [21]. In comparison our results show considerably higher values of sensitivity for fever and diarrhoea, but slightly lower values of specificity. Generally, guardians detected symptoms of common childhood illnesses with higher sensitivity but lower specificity compared to other studies [21]. Our results indicate that guardians of sick children can contribute to disease surveillance, at least by identifying their children’s symptoms.

In contrast, the results for disease assessment generally revealed inadequate performance of the tool. A possible reason may be that questions were too imprecise to achieve reliable assessment corresponding to the physicians’ findings. It was considered whether more questions could improve the assessment, but concluded that more than 15 possible items would only protract the questioning and, thus, lower user satisfaction. Moreover, one has to acknowledge the structural difference in the diagnostic process of a machine vs. a human being. The tool heavily depended on the data which were only received by asking questions; finally, data were collected in a binary yes/no form. In contrast, the physician can get additional specific clues by examining the patient and can put information into perspective.

A systematic literature search was conducted to identify mHealth studies that aimed at improving child health before the development of the algorithm. None of the reviewed publications used an algorithm-based IVR-tool for the detection of symptoms or the assessment of a child’s health status. Most of the mHealth interventions focused on improving patient follow-up and medication adherence as well as data collection/transfer and reporting [11, 22–25]. The targeted group for decision-support-systems was medical personnel [12, 16, 26–28]. The communication channels primarily used were short messaging services (SMS) [29–31], whereas IVR-systems were not used at all [7, 11, 32].

Given that this is the first study using an IVR-tool operating on a medical algorithm to receive health information and process them into a health assessment, it had several limitations that should be considered in future studies. First, the IMCI guidelines proved to be difficult when compared to the physicians’ notes so that some questions, especially the danger signs, could not be measured adequately. Reasons might be the low prevalence (e.g., for convulsions and unconsciousness, which were fast-tracked through the OPD), the lack of the guardians’ medical knowledge of danger signs (as reported in other studies [16]) or the fact that the physicians assessed a child’s health status in a different way than the IMCI. Future studies should use structured questionnaires to be filled out by the physicians during consultation to record clinical assessment and improve comparisons between the findings made by the tool and the physician.

Second, not all characteristics of the IMCI guidelines could be implemented into the algorithm. One of its key components is the assessment by the community health worker: inspecting the child (e.g., for chest indrawing), measuring the body temperature and counting the breaths (“ask, look, feel”). We could not transfer these tasks onto guardians without medical experience or otherwise the data would be unreliable and result in biased information. Thus we had to reduce the complexity of the IMCI guidelines in such a way that they could be understood by the guardians and correct data could be obtained. Naturally, this happened at the expense of the diagnostic performance, which was anticipated during the development of the algorithm. However, since we wanted to avoid recommending home care in severe illnesses, we designed the algorithm to detect symptoms as sensitive as possible and accepted the compromise between symptom detection with high sensitivity on the one hand and “overestimating” a child’s condition on the other hand.

Third, the study was conducted in a children’s OPD of a well-equipped district hospital, which implicated a higher prevalence and severity of diseases compared with everyday life. Due to this instance, the tool’s performance could be appropriately evaluated for the treatment recommendations A and B, but the prevalence of C-cases was too low. Therefore, our research group initiated another study in fieldwork to evaluate our tool in the domestic setting, where more C-cases were suspected.

The study results indicate the ability of the tool to detect symptoms of common childhood diseases and to give suitable treatment recommendations. Our research group further found that our tool was applicable and accepted by Ghanaians in focus group discussions [17]. Furthermore, the project group developed a software application for space-time surveillance and model-based analysis within the eHISS system. Provided its adequate performance in the domestic setting, the panel of clinicians and epidemiologists could use the gathered information to update and optimize the tool. In its final version, the eHISS tool is envisaged to provide people with health information and give health authorities and health policy makers reliable data on prevailing disease syndromes.

## Conclusions

This study reports the applicability of an algorithm-based IVR-tool to correctly identify symptoms of the most common childhood diseases in sub-Saharan Africa and to deliver appropriate health advice via mobile phone. While disease assessment did not work sufficiently, it was shown that symptom detection was feasible and comparable to the physicians’ findings. By providing appropriate treatment recommendations, the tool could support decision making for indecisive guardians of sick children. It could serve as a second opinion and turn the balance in favour of presenting at a hospital, while reducing the number of unnecessary journeys. Especially with distant and time-consuming trips to the closest health care facility early presentation could prevent complicated courses of disease. These study results could also be applied to enhance disease surveillance initiatives, e.g. obtaining direct information by calling people from a distinct area instead of dispatching health care personnel.

## Additional file

Additional file 1 Detailed description of the expert panel’s composition and function. This file contains detailed information about the composition of the expert panel, division of responsibilities and decision making process during the development of the algorithm and IVR-system. Additionally, the results of the panel meetings are displayed. The list of the panel members, their respective expertise and role in the project is summarized in a table. (DOCX 33 kb)

## Abbreviations

APH: Agogo Presbyterian Hospital
eHISS: electronic Health Information and Surveillance System
GI-infection: Gastrointestinal infection
IMCI: Integrated Management of Childhood Illnesses
IVR: Interactive voice response
KNUST: Kwame Nkrumah University of Science and Technology
mHealth: mobile Health
NPV: Negative predictive value
OPD: Outpatient department
PPV: Positive predictive value
RTI: Respiratory tract infection
SMS: Short message service
WHO: World Health Organization
