# Supplementary material for: A mobile phone based tool to identify symptoms of common childhood diseases in Ghana: development and evaluation of the integrated clinical algorithm in a cross-sectional study
Source: BMC Med Inform Decis Mak. 2018 Mar 27;18:23. doi: 10.1186/s12911-018-0600-3 (PMC5870385; doi:10.1186/s12911-018-0600-3)
Supplement: Supplementary file 1 — Detailed description of the expert panel’s composition and function. This file contains detailed information about the composition of the expert panel, division of responsibilities and decision making process during the development of the algorithm and IVR-system. Additionally, the results of the panel meetings are displayed. The list of the panel members, their respective expertise and role in the project is summarized in a table. (DOCX 33 kb) [file 12911_2018_600_MOESM1_ESM.docx]

## Detailed description of the expert panel's composition and function

The effectiveness of the clinical algorithm to assess disease symptoms of ill children, its translation into an interactive voice response (IVR) system and the comprehensibility of the developed tool is of significant importance to the quality of the project outcome. Hence, an expert panel was established to develop the clinical algorithm and to translate it into a user-friendly IVR system. The expert panel consisted of national and international clinical practitioners, epidemiologists/biostatisticians, public health experts, social scientists and communication researchers. Their broad range of expertise was chosen to cover the various aspects in the process to develop, evaluate and finally apply the algorithm/IVR system. Names, expertise and experience of the panel members are listed in Table S1. The different steps in the process to develop the clinical algorithm/IVR system were coordinated and conducted within respective project work packages. BeK led the development and modification of the clinical algorithm that was based on WHO’s guidelines for Integrated Management of Childhood Illness (IMCI) and the evaluation of the clinical performance of the algorithm as presented in the manuscript. JuF coordinated the evaluation of the usability of the algorithm/IVR system in a potential user group. MaB led the translation of the algorithm into an IVR system as well as its maintenance and operation.

To decide on the final clinical algorithm/IVR system, the expert panel discussed the drafted and amended versions presented during the consecutive meetings and made decisions on how to modify and improve the system. The panel group personally met three times throughout a period of two years to discuss different steps of the project. Between the three meetings the project work groups completed the tasks as agreed upon in the workshops, and the work in progress was regularly discussed among the work package leaders in telephone conferences (on average every three months). The work completed in the different groups was under responsibility of the particular work package leaders who could make final decisions for their defined tasks. In the project meetings the panel group discussed all steps concerning the development of the tool in a transparent and open manner. . To achieve this, we followed a Delphi method-like discussion approach. Project content was presented to the expert panel and discussed openly. In case no agreement on a particular procedure could be achieved, the different opinions were summarised by responsible work package leaders and presented to the panel, and participants were asked to revise their judgement. In case no agreement could be achieved, senior representatives from the participating institutions (or her/his representative) had to do a majority vote. However, the panel always achieved complete agreement during the expert discussions.

The system’s last version was presented during the final meeting and no further changes were discussed. Panel members who could not attend a meeting were informed about all discussions, to get the possibility contribute their feedback.

The following meetings were held to develop the clinical algorithm and the IVR system:

**WORKSHOP 1: 27. & 28.01.2014; BNITM, Hamburg, Germany.** Attendees: JüM, JuF, JoB, BeK, RaK, MiK, AxB, MaB, NiS. During this meeting the drafted version of the amended IMCI guidelines was presented by BeK. Panel members discussed the included and excluded symptoms and the assessment of disease severity in the light of completeness of disease symptoms, comprehensibility by lay people, ability to be translated into an IVR system and categorisation of algorithm outcome along with the corresponding clinical consequences.

**WOKSHOP 2: 30. & 31.10.2014; University of Accra, Ghana.** Attendees: ElO, JüM, JuF, JoB, KoF, RaK, AxB, PhA, AlM. The draft version of the clinical algorithm was programmed into an IVR system, which was accessible by mobile phones. This preliminary version was evaluated by JoB and JuF in four focus group discussions (FGD). In the FGD the system was explained to potential end users, the IVR system was tested by the interviewees and its pros and cons were systematically assessed. The drafted algorithm, the programmed IVR system and the algorithm outcome were discussed during the workshop. The outcome of the focus group discussions was presented and necessary adjustments were suggested and discussed. PhA presented the IVR system and highlighted programming issues that had consequences for data collection and data processing. KoF and BeK presented the results from the pilot phase of the clinical evaluation and necessary adjustments were suggested and discussed.

**WOKSHOP 3: 4. & 5.11.2015; KCCR, Kumasi, Ghana.** Attendees: ElO, JüM, JuF, JoB, BeK, RaK, AxB, PhA, AlM. The current version of the clinical algorithm and the IVR system was presented to the expert panel. The results of the focus groups discussions were highlighted in the same manner as on how this informed the adjustment of the algorithm and the IVR system. The results of the clinical evaluation of the tool as presented in the manuscript were presented and discussed. At this point no further changes were made to the established system.

**Table S1:** List of panel members with their respective expertise and role in the project.

| **Name** | **Institution** | **Field of expertise and experience** | **Role in the panel** |
| --- | --- | --- | --- |
| Dr. Ellis Owusu-Dabo* (ElO) | Kumasi Centre for Collaborative Research in Tropical Medicine (KCCR), Kumasi, Ghana | Physician, Public health  Head of the KCCR with profound expertise in translating health research into public health policies | Local coordinator of all project activities; provided valuable input for the inclusion/exclusion of symptoms, resulting advice and especially the phrasing of the algorithm in the local language. |
| Prof. Dr. Jürgen May* (JüM) | Bernhard Nocht Institute for Tropical Medicine (BNITM), Hamburg, Germany | Professor for Infectious Disease Epidemiology with >20 years of experience in research in West Africa | Project leader with input in all fields. Substantially influenced the design of the study and the final version of the algorithm |
| Prof. Dr. Julius Fobil* (JuF) | School of Public Health, University of Ghana, Accra, Ghana | Public Health and environmental health  In-depth knowledge of the local community and their health situation | Led and supervised the focus group discussions. Gave input on the design of the algorithm based on results from the focus group discussions. |
| Johanna Brinkel (JoB) | School of Public Health, University of Bielefeld, Germany | Public Health, social sciences  Trained in social sciences with extensive experience in qualitative methods | Performed and evaluated the focus group discussions. Gave input on the design of the algorithm based on results from the focus group discussions. |
| Dr. Benno Kreuels* BeK) | University Medical Center Hamburg-Eppendorf (UKE), Germany | Infectious disease consultant and epidemiologist with 10 years of experience in clinical tropical medicine and research in West Africa | Designed and presented the initial version of the algorithm. Designed and supervised the study for the evaluation of clinical performance. |
| Konstantin Franke (KoF) | UKE | Medical student | Protocolled discussions during telephone conferences and conducted the clinical evaluation as part of his MD-Thesis under the supervision of BeK. |
| Dr. Ralf Krumkamp* (RaK) | BNITM | Epidemiology, Public Health  Project coordinator, experienced infectious disease epidemiologist, working with prediction models | Coordinated the work packages and supervised the link between data collection and data analysis |
| Prof .Dr. Mirjam Kretzschmar* (MiK) | National Institute for Public Health and the Environment, Center for Infectious disease Control (RIVM) | Infectious disease modelling  Head of the Mathematical Modelling Unit of the University of Utrecht and expert in modelling surveillance data | Responsible for the evaluation of space-time associations and the established prediction models |
| Dr Axel Bonacic Marinovic (AxB) | RIVM | Biostatistics, informatics  Mathematical modeller with thorough programming skills | Responsible for the evaluation of space-time associations and the technical link between data collection and disease surveillance |
| Mark Boots* (MaB) | VOTO Mobile, Kumasi, Ghana | Informatics, communication research  Funder VOTO mobile, already implemented several health related IVR systems | Responsible for supervision concerning implementation of the algorithm into an IVR-System |
| Philip Asihene (PhH) | VOTO Mobile, Kumasi, Ghana | Informatics, communication research  advanced programmer of IVR tools | Implemented the algorithm in the IVR-system and was responsible for maintenance |
| Aliyu Mohammed (AlM) | KCCR | Public Health  health care experience with profound knowledge of the local community and health situation | Supported KoF in the evaluation of the clinical performance |
| Nimako Sarpong (NiS) | KCCR | Physician  15 years of work experience at APH and other Ghanaian hospitals and local study coordinator of several paediatric infectious disease projects | Provided valuable input for the inclusion/exclusion of symptoms, resulting advice and especially the phrasing of the algorithm in the local language. Supervised data collection during the study. |
| Dr. Teresa Rettig | Agogo Presbyterian Hospital, Ghana | Paediatrician  20 years of work experience at the paediatric ward at APH | Provided valuable input for the inclusion/exclusion of symptoms, resulting advice and especially the phrasing of the algorithm in the local language. |

* Senior representatives with voting rights in case a majority decision was needed; APH: Agogo Presbyterian Hospital
